# First complete mitochondrial genome data of *Hydrophilus bilineatus* deciphered within the genus *Hydrophilus*

**DOI:** 10.1016/j.dib.2025.111936

**Published:** 2025-08-05

**Authors:** Nakasako Juiki, Ohara Daiki, Himeyuri Sunahata, Oda Rikuto, Nagano Sotaro, Fujimoto Erina, Tamura Naoya, Takuya Kiyoshi, Shin-ya Ohba, Jun-ichi Takahashi

**Affiliations:** a Faculty of Life Science, Kyoto Sangyo University, Kamigamo, Motoyama, Kyoto 6038047, Japan; b Department of Zoology, National Museum of Nature & Science, Amakubo 4-1-1, Tsukuba 3050005, Japan; c Biological Laboratory, Faculty of Education, Nagasaki University, Bunkyo, Nagasaki 8528131, Japan

**Keywords:** Water scavenger beetle, Iriomote island, Complete mitochondrial genome, Coleoptera

## Abstract

*Hydrophilus bilineatus* (Coleoptera: Hydrophilidae) is an aquatic beetle species belonging to the family Hydrophilidae. In this study, the complete mitochondrial genome of H. bilineatus was sequenced, assembled, and annotated. The mitochondrial genome was 17,289 bp in length, with an AT content of 72.8 %. It consisted of 37 genes, including 13 protein-coding genes, 22 transfer RNA genes, and two ribosomal RNA genes. The nucleotide composition was A: 40.8 %, T: 32.0 %, C: 16.8 %, and G: 10.4 %. A phylogenetic analysis was conducted using 13 mitochondrial protein-coding genes from H. bilineatus and eight related hydrophilid species. The resulting tree is presented to show the mitochondrial gene-based relationships within Hydrophilidae.

## Graphical abstract

**Figure:**
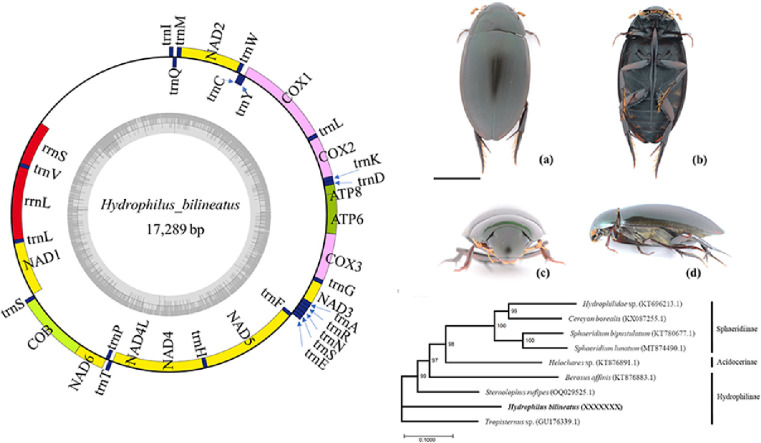

Specifications Table

**Table:** 

Subject	Biology
Specific subject area	Insecta, Mitogenome, Bioinformatics, Phylogenetic analysis
Type of data	Raw and Analyzed Table: Mitogenome data used in the phylogenetic analysis Figures: Image of *Hydrophilus bilineatus*, feature of the circular map of the *H. bilineatus* mitochondrial genome, phylogenetic tree analysis Supplementary figures: Image of the average depth of coverage, image of putative secondary structures for the tRNA genes of the *H. bilineatus* mitochondrial genome
Data collection	Genomic DNA source: An individual specimen Genomic DNA isolation: DNeasy Blood and Tissue Kit (Qiagen, Hilden, Germany) Library construction: KAPA HyperPrep Set for Illumina (NIPPON Genetics, Tokyo, Japan) Sequencing: Illumina MiSeq platform (300 bp pair end; Illumina, San Diego, CA, USA) Quality check: Illumina Experiment Manager, MiSeq Reporter Assembly and annotation: Genious 9, MITOS2 webserver Mitogenome map construction: OrganellarGenomeDRAW Phylogenetic tree analysis: MEGA v.11.0 PCGs composition analysis: Genetyx 15 tRNAs structure prediction: tRNAscan-SE webserver Phylogenetic tree visualization: MEGA v.11.0
Data source location	Location: Irimote Island (37°25′N, 126°55′E) City: Taketomi Town Country: Japan
Data accessibility	Repository name: DNA Data Base of Japan (DDBJ) DDBJ Accession number: LC875518 Bioproject number: PRJDB20545 Direct URL to data: https://getentry.ddbj.nig.ac.jp/getentry/na/LC875518/ https://ddbj.nig.ac.jp/search/entry/bioproject/PRJDB20545
Related research article	Not applicable

## 1 Value of the Data

• This study provides the first complete mitochondrial genome sequence of a species in the *Hydrophilus* genus, contributing to taxonomy, phylogenetics, and DNA barcoding.
• These data will facilitate the development of DNA markers to assess genetic diversity and gene flow. Understanding the genetic structure of natural populations may aid conservation efforts for *H. bilineatus* and related species.
• Complete mitochondrial genome data allow improved statistical reliability and resolution in species identification and phylogenetic classification compared with partial gene sequences. Furthermore, this study updates the genetic records of *H. bilineatus* in Japan and enhances public genetic databases.

## 2 Background

**Fig. 1:**
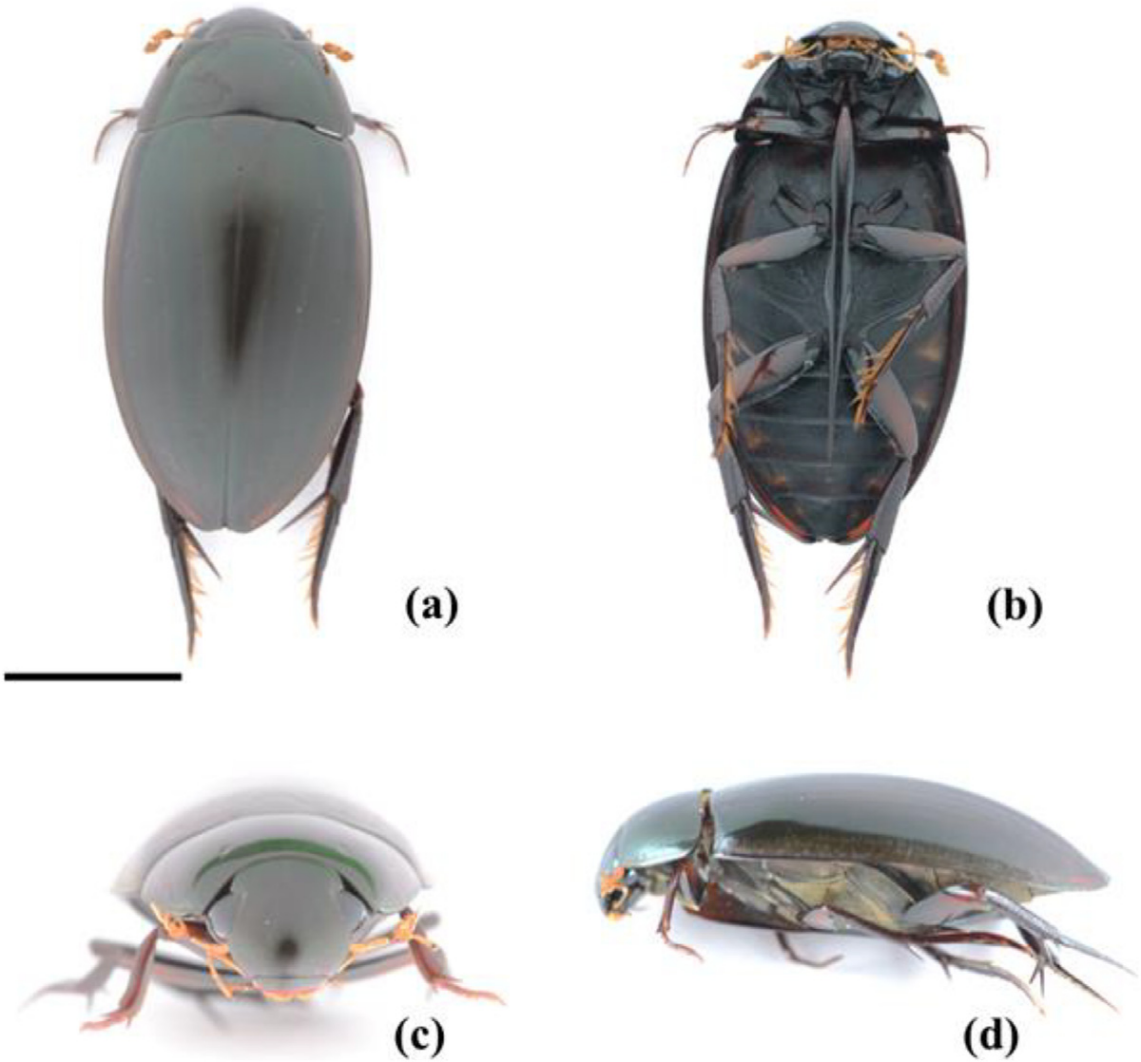
An adult water scavenger beetle, *Hydrophilus bilineatus*, was obtained from Iriomote Island, Japan. (a) Dorsal view, (b) Ventral view, (c) Head, (d) Lateral view. Scale bar = 10 mm.

*Hydrophilus bilineatus* (Fig. 1) is an important environmental indicator species of freshwater ecosystems in Japan [1]. Ecologically, it decomposes, plant and animal matter, thereby contributing to nutrient cycling and freshwater ecosystem maintenance. However, this species faces multiple threats, including habitat destruction, invasiveness, and pesticide pollution, leading to a significant population decline. Despite the ecological importance of water scavenger beetles, previous research has primarily focused on indoor breeding. No studies have investigated their genetic diversity or gene flow, —both of which are essential aspects for conservation. Currently, only partial sequences of the mitochondrial *COX1* gene of *H. bilineatus* are available in GenBank, with only four registered DNA barcoding sequences. Although the *Hydrophilus* genus comprises approximately 50 species [2], no full-length mitochondrial genome has been sequenced for any species in this group. To address this gap, we sequenced and characterized the complete mitochondrial genome of the Japanese water scavenger beetle *H. bilineatus*, marking a crucial first step in molecular phylogenetic research toward the development of DNA markers for conservation purposes.

## 3 Data Description

Sequencing generated 801,450 paired-end reads, totaling 240,435,000 base pairs. The proportion of high-quality bases (*Q* ≥ 30) was 85.4 %. The complete mitochondrial genome of *Hydrophilus bilineatus* was 17,289 bp in length (GenBank accession number: LC875518). The nucleotide composition was A: 40.8 %, C: 16.8 %, T: 32.0 %, and G: 10.4 %. The AT and GC contents were 72.8 % and 27.2 %, respectively. The AT skew and GC skew values were 0.122 and –0.236, respectively. Mitochondrial genome assembly was obtained with an average sequencing depth of 384 (Supplementary Fig. 1).

**Fig. 2:**
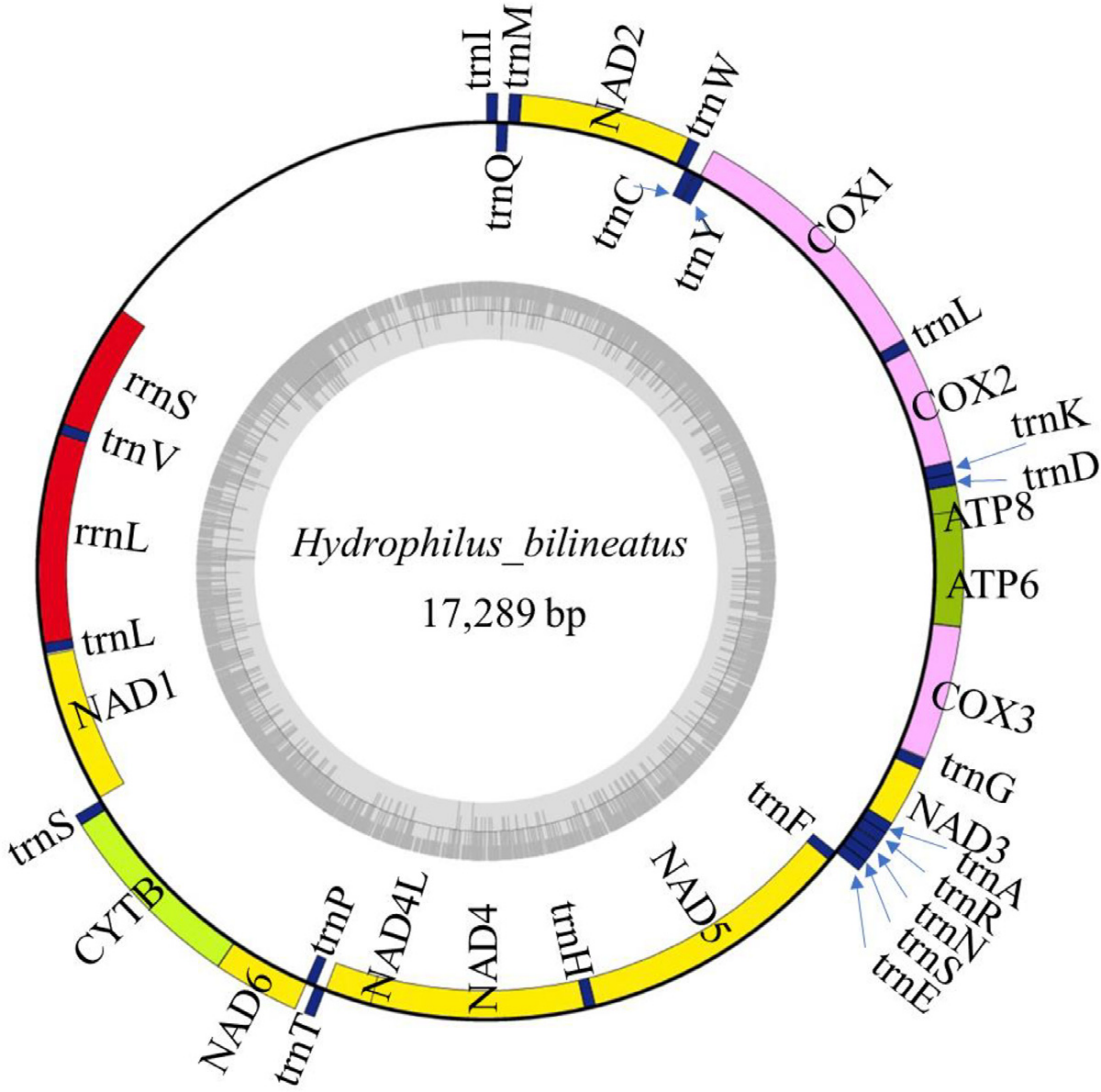
Physical map of the mitochondrial genome of *Hydrophilus bilineatus*. The legend depicts the gene type using colors: protein-coding genes: *COX1, COX2*, and *COX3* in pink; *ATP6* and *ATP8* in green; *NAD1, NAD2, NAD3, NAD4, NAD4L, NAD5*, and *NAD6* in yellows; *COB* in yellow; transfer RNA (tRNA) in blue; and ribosomal RNA (rRNA) in red. Arrows indicate gene orientation.

The annotated mitochondrial genome contained a total of 37 genes, including 13 protein-coding genes (PCGs), 22 tRNA genes, two rRNA genes, and one control region (Fig. 2). Among these, ten genes (four PCGs: *nad1, nad4, nad4L, nad5*; six tRNAs: *trnQ, trnC, trnY, trnF, trnP, trnL1*) were encoded on the complementary strand (Table 1). The mitochondrial genome included 10 intergenic spacer regions ranging from 1 to 17 bp in length, with the longest located between *trnS2* and *nad1*. Sixteen overlapping regions were identified, ranging from 1 to 64 bp, the longest of which occurred between *trnL* and the large ribosomal RNA gene (*l-rRNA*, 16S).

**Table 1:** Annotation of the *Hydrophilus bilineatus* mitochondrial genome.

Feature	Strand	Position		Length (bp)	Intergenic nucleotide (bp)	Codon		
						Anti	Start	Stop
tRNA I	+	3	68	66	−4	GAT		
tRNA Q	-	65	134	70	0	TTG		
tRNA M	+	134	203	70	2	CAT		
NAD2	+	204	1205	1002	0		ATC	TAA
tRNA W	+	1205	1275	71	−9	TCA		
tRNA C	-	1267	1331	65	4	GCA		
tRNA Y	-	1334	1400	67	−36	GTA		
COX1	+	1365	2931	1567	2		ATT	TAA*
tRNA L2	+	2932	2998	67	0	TAA		
COX2	+	2998	3685	676	2		ATT	TAA*
tRNA K	+	3686	3757	72	−3	CTT		
tRNA D	+	3755	3822	68	0	GTC		
ATP8	+	3822	3977	156	−6		ATT	TAA
ATP6	+	3971	4645	675	0		ATG	TAA
COX3	+	4645	5433	789	5		ATG	TAA
tRNA G	+	5437	5502	66	−4	TCC		
NAD3	+	5499	5855	357	−1		ATT	TAG
tRNA A	+	5854	5922	69	−4	TGC		
tRNA R	+	5919	5985	67	3	TCG		
tRNA N	+	5987	6053	67	0	GTT		
tRNA S1	+	6053	6120	68	0	TCT		
tRNA E	+	6120	6186	67	−3	TTC		
tRNA F	-	6184	6251	68	−18	GAA		
NAD5	-	6234	7967	1734	2		ATT	TAA
tRNA H	-	7968	8033	66	0	GTG		
NAD4	-	8033	9368	1336	−7		ATG	TAA*
NAD4L	-	9362	9652	291	4		ATG	TAA
tRNA T	+	9655	9720	66	−2	TGT		
tRNA P	-	9719	9783	65	−9	TGG		
NAD6	+	9775	10,284	510	0		ATT	TAA
COB	+	10,284	11,426	1143	−2		ATG	TAG
tRNA S2	+	11,425	11,490	66	18	TGA		
NAD1	-	11,507	12,448	942	11		ATG	TAG
tRNA L1	-	12,458	12,523	65	−64	TAG		
L-rRNA	-	12,460	13,829	1370	−9			
tRNA V	-	13,821	13,890	70	−3	TAC		
S-rRNA	-	13,888	14,677	790				

⁎ TAA stop codon is completed by the addition of 3′ A residues to the mRNA.

**Fig. 3:**
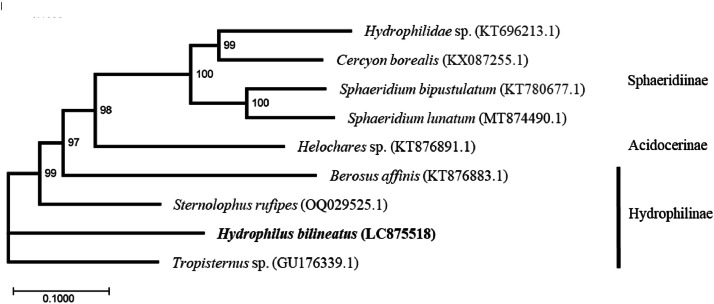
Phylogenetic relationships (maximum likelihood) of *Hydrophilus bilineatus*, Sphaeridiinae, Acidocerinae and Hydrophilinae (family Hydrophilidae) based on the nucleotide sequence of the 13 protein-coding genes in the mitochondrial genome. Numbers beside each node represent the percentages of 1000 bootstrap values. Alphanumeric terms in parentheses indicate GenBank accession numbers as follows: *Hydrophilidae* sp. (KT696213.1), *Cercyon borealis* (KX087255.1), *Sphaeridium bipustulatum* (KT780677.1), *Sphaeridium lunatum* (MT874490.1), *Helochares* sp. (KT876891.1), *Berosus affinis* (KT876883.1), *Sternolophus rufipes* (OQ029525.1), and *Hydrophilus bilineatus* (LC875518). *Tropisternus* sp. (GU176339.1) was used as an outgroup.

All 13 PCGs used ATN as the start codon. Specifically, six genes (*COX1, COX2, ATP8, NAD3, NAD5, NAD6*) started with ATT; six genes (*ATP6, COX3, NAD4, NAD4L, COB*) with ATG; and one (*NAD2*) with ATC. Stop codons varied: *ATP6, ATP8, COX3, NAD2, NAD4L, NAD5*, and *NAD6* terminated with TAA; *NAD1, NAD3*, and *CYTB* with TAG; and *COX1, COX2*, and *NAD4* had incomplete stop codons (T), which are typically completed by post-transcriptional polyadenylation. The 22 tRNA genes ranged in size from 65 to 72 bp. All tRNAs formed the typical cloverleaf secondary structure, except *trnI, trnE*, and *trnS1*, which lacked the dihydrouridine (DHU) arm. The small ribosomal RNA (*s-rRNA*, 12S) and large ribosomal RNA (*l-rRNA*, 16S) genes were 790 bp and 1370 bp in length, respectively, and were separated by *trnV* (Supplementary Fig. 2 and Table S1). Pairwise comparisons of the 13 PCGs with those of eight related species from GenBank [[3], [4], [5], [6], [7]] were conducted using MEGA X. Maximum likelihood (ML) analysis was also performed using all 13 PCGs (Fig. 3).

## 4 Experimental Design, Materials and Methods

### 4.1 Sample collection

The *H. bilineatus* specimen was collected on November 25, 2020, on Iriomote Island, Japan (24°23′15″N, 123°44′50″E). The individual, attracted to streetlights, was found dead after a traffic accident. The samples were immediately preserved in 99 % ethanol [8] and transported to the laboratory. The specimens were deposited in the National Museum of Nature and Science, Japan (voucher number NSMT-I-C-200366, https://www.kahaku.go.jp, togodb_info@kahaku.go.jp).

### 4.2 DNA extraction and NGS sequencing

The hind legs of *H. bilineatus* were used for DNA extraction. Total DNA was immediately extracted using a DNeasy Blood and Tissue Kit (Qiagen). Complete mitochondrial DNA was sequenced using a MiSeq Next-Generation Sequencer (Illumina). DNA was quantified using a Synergy LX microplate reader (Agilent Technologies) and QuantiFluor dsDNA System (Promega). Library preparation was conducted using the KAPA HyperPrep Set for Illumina (NIPPON Genetics) following the manufacturer’s instructions. The preparation conditions included an input DNA amount of 50 ng, enzymatic fragmentation time of 8 min, and seven PCR cycles. A Qubit 3.0 Fluorometer and dsDNA HS Assay Kit (Thermo Fisher Scientific) were used to determine the concentration of the prepared library solution, which was 23.9 ng/µL in a total volume of 30 µL. Library quality assessment was carried out using a Fragment Analyzer and dsDNA 915 Reagent Kit (Agilent Technologies). Sequencing was performed using MiSeq (Illumina) using 2 × 300 bp paired-end reads.

The complete mitochondrial genome of *Sternolophus rufipes* (OQ029525.1) was used as the reference sequence to assemble the reads using the bioinformatics software Geneious R9 [9]. Complete mitochondrial DNA sequences were annotated using MITOS2 [10] on the Galaxy Web server (https://usegalaxy.org). The secondary structure of tRNA was predicted using tRNAscan [11]. The AT content and codon usage were calculated using Geneious R9. The 13 PCG sequences were aligned using Genetyx ver.15 (GENETYX).

### 4.3 Phylogenetic analysis

Phylogenetic analysis was conducted using 10,803 bp of 13 mitochondrial PCGs of *H. bilineatus* (generated in this study) and seven related taxa, with sequence data for the latter obtained from the NCBI GenBank database. *Tropisternus* sp. (GU176339.1) was designated as the outgroup. The analysis was performed using the ML method in MEGA XI [12]. Sequences were partitioned by gene and codon positions, and the general time-reversible model with gamma-distributed rate variation among sites was identified as the optimal model for sequence evolution based on the Akaike Information Criterion. Bootstrap support values exceeding 90 % were considered statistically significant, with 1000 replicates used.

## Limitations

Not applicable.

## Ethics Statement

The authors have read and followed the ethical requirements for publication in Data in Brief and confirmed that the current work does not involve human subjects, animal experiments, or any data collected from social media platforms.

## Credit Author Statement

**J. Nakasako:** conceptualization, investigation, data curation, writing; **D. Ohara:** methodology, data curation; **H. Sunahata:** investigation, data curation; **R. Oda:** investigation; **S. Nagano:** data curation; **E. Fujimoto:** data curation; **N. Tamura:** investigation, writing; **T. Kiyoshi:** review & editing; **S. Ohba:** funding acquisition, review & editing; **J. Takahashi:** conceptualization, validation, supervision, review & editing.

## Data Availability

Hydrophilus bilineatus NSMT:I-C-200366 mitochondrial DNA, complete genome (Original data)
Conservation Genetic Analysis of Endangered Aquatic Insects (Original data)

## Appendix Supplementary materials

Figure S1. Overall coverage depth of the mitochondrial DNA assembly of *Hydrophilus bilineatus*. Image, application 1
