# Supplementary figures and images for: First complete mitochondrial genome data of Hydrophilus bilineatus deciphered within the genus Hydrophilus
Source: Data Brief. 2025 Aug 5;62:111936. doi: 10.1016/j.dib.2025.111936 (PMC12356376; doi:10.1016/j.dib.2025.111936)

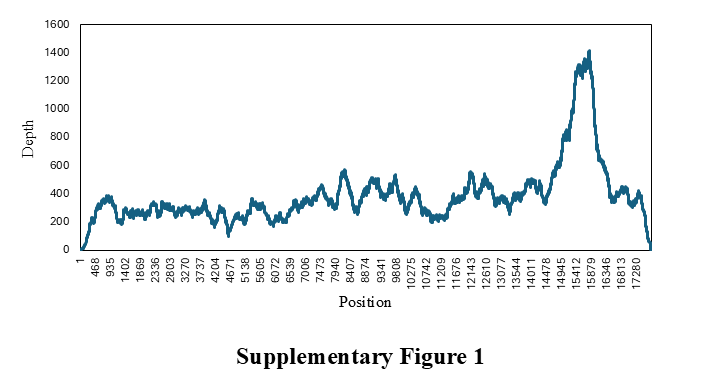


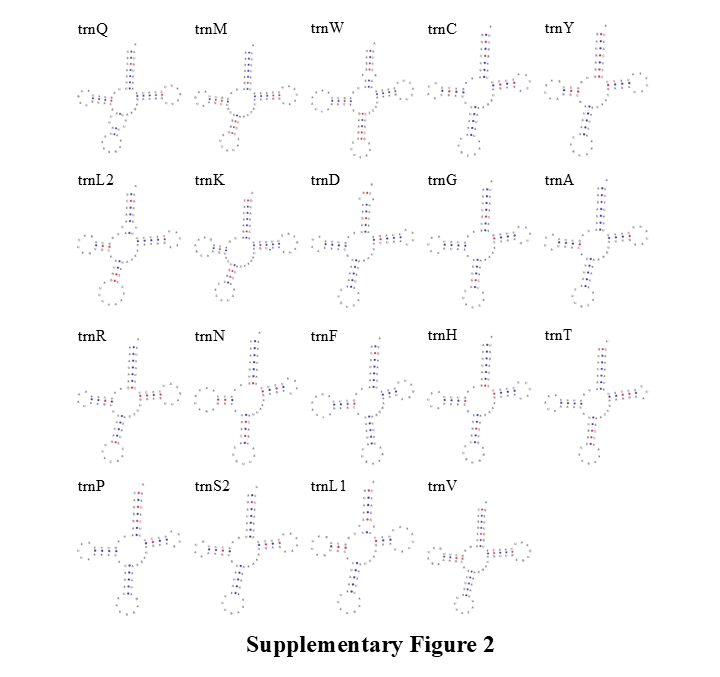

Supplement: Supplementary file 1 — Figure S1. Overall coverage depth of the mitochondrial DNA assembly of Hydrophilus bilineatus. [file mmc1.docx]
